# Cutaneous stigmata indicative of occult spinal dysraphism in two ancient Roman statues

**DOI:** 10.1007/s00381-026-07258-0

**Published:** 2026-04-22

**Authors:** Kathleen Bubb, Yoko Tabira, Carmine Antonio Donofrio, Filippo Badaloni, Antonio Fioravanti, Joe Iwanaga, R. Shane Tubbs

**Affiliations:** 1 Division of Anatomy, Department of Radiology, Weill Cornell Medicine, New York, NY USA; 2 Division of Gross and Clinical Anatomy, Department of Anatomy, Kurume University School of Medicine, 67 Asahi-Machi, Kurume, Fukuoka Japan; 3 Department of Neurosurgery, ASST Cremona, Cremona, Italy; 4 Department of Neurosurgery, IRCCS Istituto Delle Scienze Neurologiche Di Bologna, Bologna, Italy; 5 Department of Neurosurgery, Tulane Center for Clinical Neurosciences, Tulane University School of Medicine, 131 S. Robertson St., Suite 1300, New Orleans, LA 70112 USA; 6 Department of Neurology, Tulane Center for Clinical Neurosciences, Tulane University School of Medicine, New Orleans, LA USA; 7 Department of Structural & Cellular Biology, Tulane University School of Medicine, New Orleans, LA USA; 8 Department of Surgery, Tulane University School of Medicine, New Orleans, LA USA; 9 Department of Neurosurgery and Ochsner Neuroscience Institute, Ochsner Health System, New Orleans, LA USA; 10 University of Queensland, Brisbane, Australia; 11 Department of Otolaryngology, Tulane University School of Medicine, New Orleans, LA USA

**Keywords:** Spine, Spina bifida occulta, Ancient, Antiquity, History, Art

## Abstract

Symptoms of tethered spinal cord syndrome often present later in life and go undiagnosed due to a lack of appreciation of the associated cutaneous stigmata. This is especially true with occult spinal dysraphism. Here, we report cutaneous signs of occult spinal dysraphism identified in two ancient Roman sculptures. Although the significance of such findings may not have been realized, the association between cutaneous stigmata of the back and underlying malformations involving the spinal cord is now recognized, as such findings are typically imaged to evaluate for spinal cord tethering.

## Introduction

Spinal dysraphism or dysraphic states are defects in caudal neural tube formation and are classified as open or “aperta” and closed or “occulta” [1]. Occult spinal dysraphism (OSD) encompasses a range of midline neural tube defects (NTDs) in which the lesion is covered by skin [2, 3]. Such OSDs include lipomyelomeningoceles, and neurenteric and dermoid cysts [1, 4–6]. Others, like split cord malformations, congenital dermal sinuses and tracts, and meningocele manqué, are not commonly associated with subcutaneous swellings [7]. Although these lesions have different developmental mechanisms, they all result in some form of spinal cord tethering [5]. The exact prevalence of OSDs is unclear because many patients may not show symptoms. However, OSDs are usually accompanied by midline cutaneous stigmata [8]. A study by Choi et al. reported that OSD occurred in 2.8% of patients with cutaneous stigmata of the back. Such skin lesions accompanying OSDs range from focal hirsutism [4], flat capillary hemangiomas [3], aplastic and pigmented spots, skin tags, and appendages [6], and coccygeal pits. Further, although rare, some lumbosacral dimples are associated with OSDs [9, 10]. When present, dimpling linked to OSD is found cranial to the intergluteal cleft and outside the midline, unlike benign sacral dimples like coccygeal pits found in the midline and within the cleft [8, 11). Focal midline hirsutism is a recognized cutaneous marker of occult spinal dysraphism and may be associated with split cord malformation. Herein, we describe two ancient Roman sculptures with cutaneous stigmata that could represent OSD.

### Case illustrations

#### Case 1

**Fig. 1:**
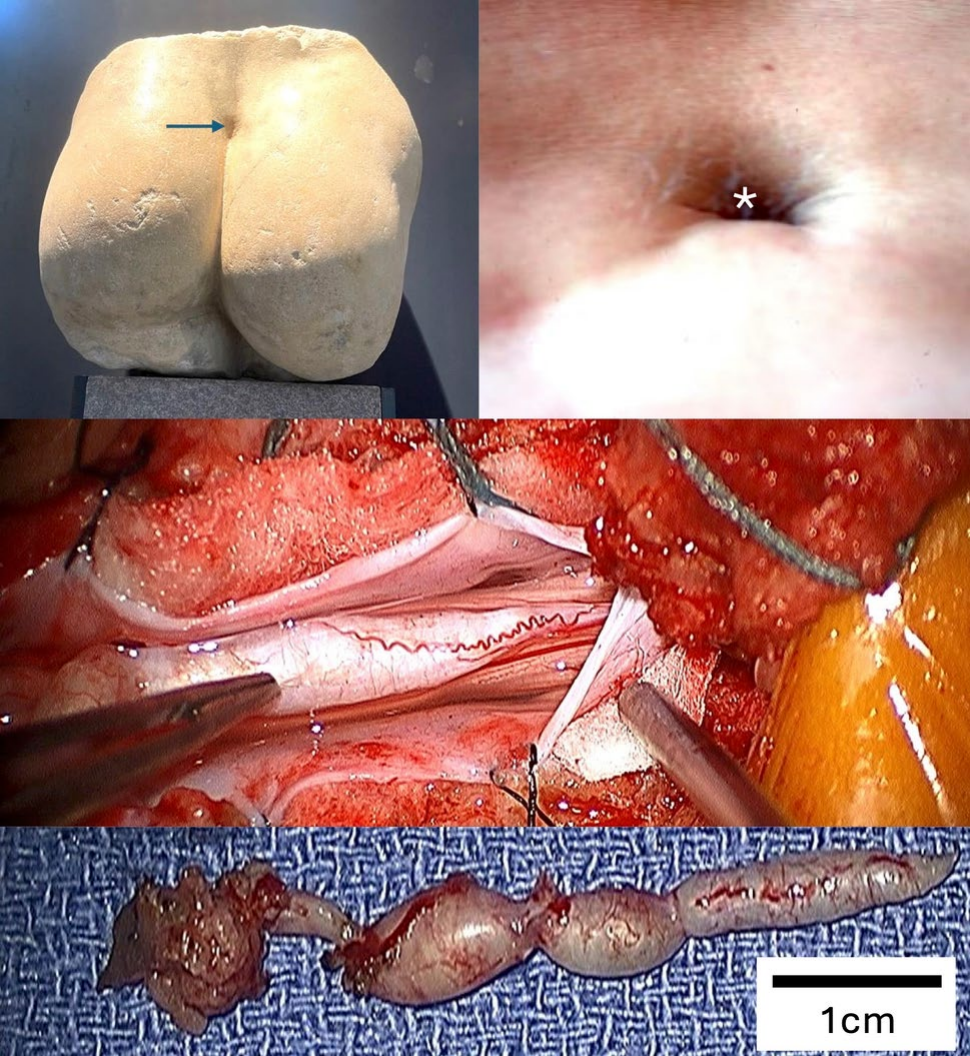
Case 1. Sculptured white marble (upper left) representation of the posterior aspect of a human torso (*parte posteriore di sculptura*) discovered in the vicinity of the Palazzo Comunale (Town Hall) in Cremona, Italy, and dated to the 1st–2nd century A.D. The work is currently housed at the Museo Archeologico di San Lorenzo, Cremona, Italy. The sculpture displays an elliptical midline defect (arrow) in the lumbosacral region, located immediately superior to the intergluteal cleft, with longitudinal and transverse diameters of approximately 4 cm and 5 cm, respectively. The borders of the defect are smooth and sharply defined, with a shallow depression suggesting deliberate carving rather than accidental breakage or erosion. Fine tool marks and surface weathering indicate age-related patina rather than modern alteration. A subtle asymmetry of the gluteal musculature—notably, attenuation of the left gluteal contour relative to the right—is discernible, possibly intended to accentuate the defect’s prominence. The configuration and position of this midline depression raise the possibility that the artist represented a developmental midline anomaly or lesion suggestive of a spinal dysraphic feature, such as a split cord malformation, myeloschisis, or dermal sinus tract, rather than an incidental surface irregularity. This may reflect either an intentional anatomical observation, a symbolic depiction of vulnerability or divinity, or a representation of a known congenital condition within Greco-Roman sculptural realism. Also note a dermal sinus in a similar location in a patient (*) (upper right), surgical exposure of this patient’s dermal sinus tract, which entered the caudal spinal cord (middle image) and the resected dermal sinus tract (lower image)

Case 1 is a sculptured white marble representation of the posterior aspect of the human torso (*parte posteriore di sculptura*) discovered in the vicinity of the Palazzo Comunale (Town Hall) in Cremona, Italy, and dated to the first–second century A.D. The work is presently housed in the San Lorenzo Museo Archeologico, Cremona. Examination of the statue reveals an elliptical midline defect in the lumbosacral region, situated immediately above the superior margin of the intergluteal cleft, with longitudinal and transverse diameters measuring approximately 4 cm and 5 cm, respectively. The defect is sharply marginated and surrounded by subtle surface irregularity consistent with a sculpted representation rather than a post-depositional fracture. A slight asymmetry of the gluteal contours—with attenuation of the left gluteal mass relative to the right—is also apparent. Taken together, the morphology and topography of this defect suggest that the artist may have intentionally depicted an anatomic anomaly or lesion in the lower spinal region, potentially representing a congenital dorsal defect such as a split cord malformation or a related dysraphic condition, rather than an incidental surface imperfection (Fig. 1).

#### Case 2

Case 2 is a marble sculpture (Roman copy of a Greek original from the fourth century B.C.; first–second century A.D.) depicting a satyr carrying the infant Dionysus, currently preserved in the Vatican Museo Pio-Clementino. On detailed inspection of the posterior surface, a zone of focal hirsutism—or “faun’s tail” configuration—is evident along the mid- to upper-lumbar midline. The modeled hair pattern is highly localized and vertically oriented, distinct from the surrounding surface texture, suggesting deliberate artistic intent rather than a generalized depiction of body hair. The anatomical position of the hair tuft corresponds to the classical cutaneous hallmark of occult spinal dysraphism, often clinically described as a “faun tail” sign in patients with an underlying OSD such as split cord malformation. Whether the sculptor sought to symbolize the satyr’s hybrid nature or inadvertently represented a realistic congenital feature remains open to interpretation. Yet, its midline position and focal distribution are of particular clinical-anatomical interest.

## Discussion

We show two sculptures from Roman antiquity that demonstrate cutaneous stigmata of OSD, a dermal sinus, and focal hirsutism.

### Dermal sinuses

Dermal sinuses (DSs) and dermal sinus tracts (DSTs) are one form of OSD characterized by small openings along the spine, believed to result from incomplete separation of the neuroectoderm from the embryonic ectoderm, which maintains an epidermal-lined connection or tract between the skin and neural elements (Ackerman & Menezes, 2003). Although DS-type lesions can develop anywhere along the midline, they are commonly found as small openings in the lumbosacral region above the intergluteal cleft. They are frequently associated with other cutaneous stigmata of OSD, such as focal hirsutism (hypertrichosis), flat capillary hemangiomas, and skin discoloration [11]. While DSTs may appear innocuous, they can pose significant health risks to patients if left untreated [12, 13]. It follows that these lesions should be evaluated by ultrasound and MRI, and that surgical exploration of DSs is warranted, as the epidermal-lined tracts tend to connect to neural or spinal abnormalities cranial to the sinus, including tethered cord, inclusion tumors, and split cord malformations [14]. The epidermis-lined tracts are potential pathways for central nervous system infections, with the most frequently presenting symptoms in patients with DS/DST defects. The most common causes for referral were abnormal skin findings (57.1%) and infection (31.4%) [10]. Dermal sinus tracts can be associated with the drainage of spinal fluid, intradural dermoid or epidermoid cysts, and spinal cord tethering. Although around 25% of sacral DSs seen at birth may develop into a deep dimple over time, all dermal sinuses should be surgically explored and treated before any neurological symptoms or signs of infection appear [9, 12].

### Focal hirsutism

Although not pathognomonic, focal hirsutism, especially of the midline, can be associated with an underlying split cord malformation. Split cord malformations (SCMs) are a form of occult spinal dysraphism (OSD) characterized by a longitudinal division of the spinal cord into two hemicords, each containing its own central canal and posterior and anterior nerve roots [15]. This division may occur over a variable length of the spinal axis and is typically classified into two main types based on the presence or absence of a duplicated dural sheath. Type I SCMs consist of two hemicords housed within separate dural tubes, separated by a rigid osseocartilaginous septum, while type II SCMs have both hemicords enclosed within a single dural sac, divided by a fibrous septum [15, 16].

The condition is thought to arise during early embryogenesis, specifically due to abnormal adherence between ectoderm and endoderm, leading to the formation of an accessory neurenteric canal and subsequent splitting of the notochord [17]. This embryologic defect results in the formation of an abnormal median septum that divides the developing neural tube. SCMs can occur at any spinal level but are most frequently located in the thoracolumbar or lumbar regions [15]. They are commonly associated with other spinal anomalies such as tethered cord, lipomas, dermal sinuses, or meningoceles. They may also present alongside cutaneous manifestations, including focal hirsutism, capillary hemangiomas, or skin dimples [11].

Clinically, SCMs may present with neurological deficits such as lower limb weakness, gait disturbances, or scoliosis, and may be discovered during evaluation for cutaneous stigmata of OSD [14]. Management typically involves surgical correction to excise the septum and detether the spinal cord to prevent or halt progression of neurological symptoms [15]. Early recognition and intervention are essential to reduce the risk of irreversible neurological deterioration.

Surgical outcomes are generally favorable when performed before a significant neurological decline. Therefore, as with other OSD lesions, such as dermal sinus tracts, prompt surgical evaluation and management are warranted to prevent complications and preserve neurological function [12].

### Depictions of congenital malformations in ancient art across cultures

**Fig. 2:**
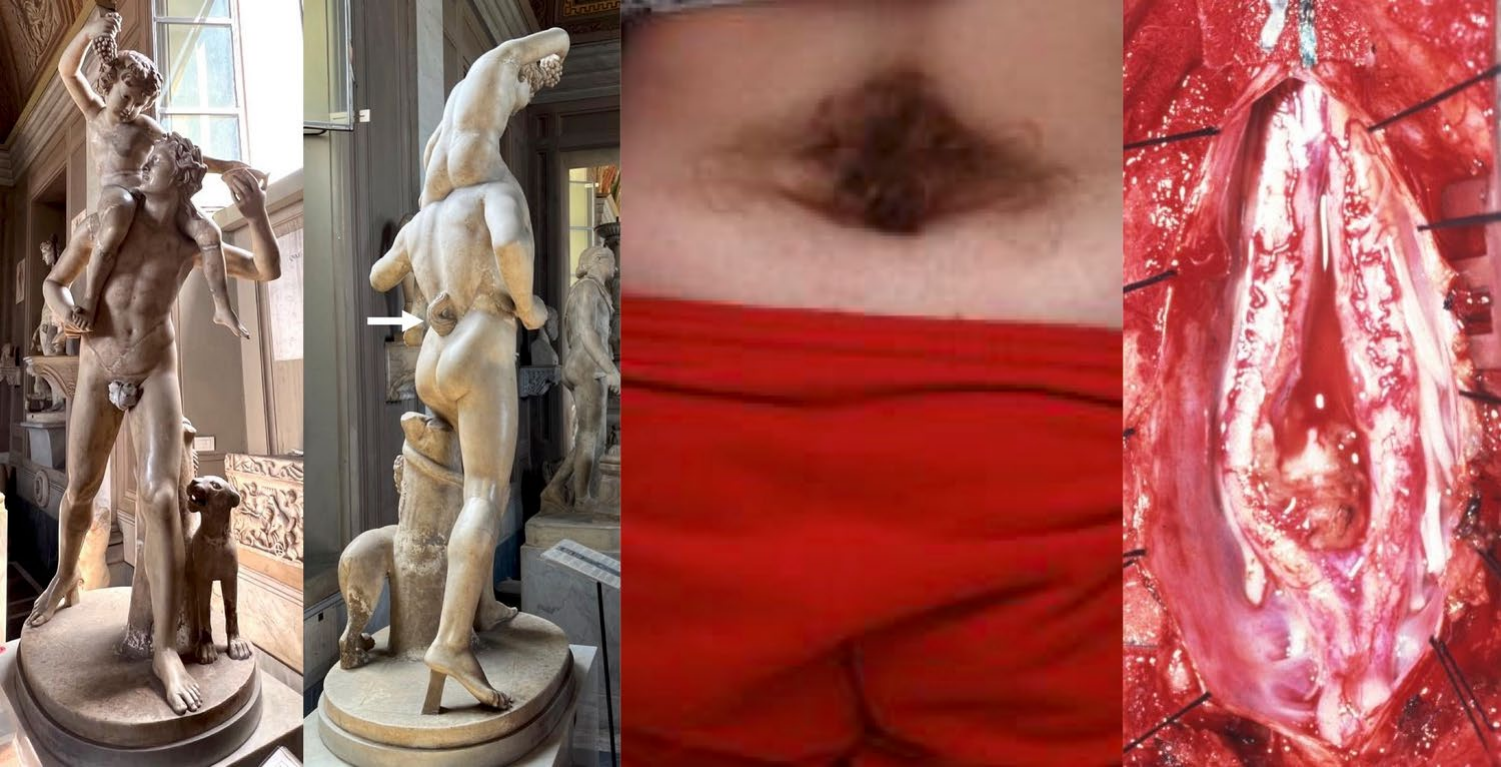
Case 2. Marble sculpture (left image) (Roman copy of a Greek original from the 4th century B.C.; 1st–2nd century A.D.) depicting a satyr carrying the infant Dionysus, currently preserved in the Vatican Museo Pio-Clementino. Upon close examination of the posterior surface, a distinct focal hirsutism or “faun’s tail” (arrow) configuration is observed along the mid- to upper-lumbar midline. The localized vertical modeling of the hair stands out in relief from the adjacent smooth marble surface, differing in both texture and depth from the generalized rendering of body hair elsewhere on the figure. This concentrated zone of sculpted hair conveys a striking resemblance to the cutaneous stigmata of occult spinal dysraphism, most notably the “faun tail sign” described in modern neurosurgical literature as an indicator of underlying tethered cord or split cord malformation. While the feature could reflect the mythological satyr’s hybrid nature—part human, part animal—it also evokes a clinically accurate representation of midline focal hypertrichosis, a finding rarely paralleled in ancient art. The precision and anatomical correspondence suggest that the sculptor may have drawn from direct human observation rather than mythic imagination, making this figure of exceptional relevance to the intersection of classical art and medical iconography. The middle image shows a patient with midline, lumbosacral focal hirsutism. The right image is the same patient at surgery, noting a split cord malformation

Depictions of congenital malformations can be found in ancient sculptures and other forms of art. For example, achondroplasia and other forms of dwarfism are portrayed as divinities in the art of ancient Egypt and Greece, as well as in pre-Hispanic cultures of Central and South America (Fig. 2) [18]. The elusive merfolk may be a case of symelia, and they were first depicted in the artwork of multiple pre-Columbian native American cultures as early as 600 BC[19]. Orticochea [20] described a lateral cleft in 2000-year-old pottery from the Bahia culture on the Pacific coast of South America. In a 2019 case report, Deps and Charlier [21] diagnosed Crouzon syndrome, a rare congenital craniofacial disorder, in 3,000-year-old Mayan pottery. Similarly, Goodrich and Ponce de Leon [22] identified multiple cases of open spinal dysraphism, including meningomyelocele, in terra-cotta artwork from several Mesoamerican civilizations. As mentioned above, Williams and Levitt [23] proposed that the tail in Hellenistic sculptures of the Greek god Pan is likely OSD-associated hypertrichosis. In fact, hypertrichosis is one of the most common cutaneous signs of OSDs and is depicted in artwork from multiple ancient cultures [6, 23]. The model for the sculpture was likely an adult with no other cutaneous signs of OSDs, but in the absence of the thorax and limbs, it cannot be confirmed that there were symptoms of cord tethering. However, the midline pinpoint lesion above the intergluteal cleft might suggest a dermal sinus. Additionally, as satyrs were often depicted with exaggerated hair patterns reflecting their hybrid nature, it is possible that the sculpture in our case illustration represents this rather than a cutaneous stigmata of OSD. This would be supported by the fact that this statue was not shown to have any additional signs of a tethered spinal cord such as limb atrophy/asymmetry.

## Conclusion

The two illustrative cases reported here could be unrelated artistic depictions with no association to an underlying OSD. However, without additional information, one or both examples might be related to pathology and at least serve as materials for conversation, such as other reports of pathologies depicted in art as mentioned above.

## Data Availability

No datasets were generated or analysed during the current study.
